# The phytochemical polydatin ameliorates non‐alcoholic steatohepatitis by restoring lysosomal function and autophagic flux

**DOI:** 10.1111/jcmm.14320

**Published:** 2019-04-11

**Authors:** Xiaoting Chen, Hung Chan, Lin Zhang, Xiaodong Liu, Idy H. T. Ho, Xiang Zhang, Jeffery Ho, Wei Hu, Yuanyuan Tian, Shanglong Kou, Chee Sam Chan, Jun Yu, Sunny H. Wong, Tony Gin, Matthew T. V. Chan, Xuegang Sun, William K. K. Wu

**Affiliations:** ^1^ School of Traditional Chinese Medicine Southern Medical University Guangzhou People’s Republic of China; ^2^ Zhujiang Hospital, Southern Medical University Guangzhou People’s Republic of China; ^3^ Department of Anaesthesia and Intensive Care The Chinese University of Hong Kong Shatin Hong Kong; ^4^ State Key Laboratory of Digestive Diseases Li Ka Shing Institute of Health Sciences, The Chinese University of Hong Kong Shatin Hong Kong; ^5^ CUHK Shenzhen Research Institute Shenzhen People’s Republic of China; ^6^ Department of Medicine & Therapeutics The Chinese University of Hong Kong Shatin Hong Kong

**Keywords:** cathepsin D, LC3, lipophagy, NAFLD, p62

## Abstract

Impaired autophagic degradation of intracellular lipids is causally linked to the development of non‐alcoholic steatohepatitis (NASH). Pharmacological agents that can restore hepatic autophagic flux could therefore have therapeutic potentials for this increasingly prevalent disease. Herein, we investigated the effects of polydatin, a natural precursor of resveratrol, in a murine nutritional model of NASH and a cell line model of steatosis. Results showed that oral administration of polydatin protected against hepatic lipid accumulation and alleviated inflammation and hepatocyte damage in *db*/*db* mice fed methionine‐choline deficient diet. Polydatin also alleviated palmitic acid‐induced lipid accumulation in cultured hepatocytes. In both models, polydatin restored lysosomal function and autophagic flux that were impaired by NASH or steatosis. Mechanistically, polydatin inhibited mTOR signalling and up‐regulated the expression and activity of TFEB, a known master regulator of lysosomal function. In conclusion, polydatin ameliorated NASH through restoring autophagic flux. The polydatin‐regulated autophagy was associated with inhibition of mTOR pathway and restoration of lysosomal function by TFEB. Our study provided affirmative preclinical evidence to inform future clinical trials for examining the potential anti‐NASH effect of polydatin in humans.

## 1 INTRODUCTION

Non‐alcoholic fatty liver disease (NAFLD) is a chronic liver disease that encompasses a spectrum of hepatic pathologies, from simple hepatic steatosis, through hepatic steatosis with lobular inflammation, to non‐alcoholic steatohepatitis (NASH).^1^ The prevalence of NAFLD has increased from less than 10% in the 1980s to the current rate of 15%‐30% and will become the most common chronic liver disease in the future.^2^ The accumulating evidence indicated that lipotoxicity contributes to the current paradigm of NASH through complex molecular signalling pathways, involving overproduction of free radicals from the mitochondria, causing lipid peroxidation, cytokine production and necrosis.^3^

Autophagy is a regulated intracellular degradation system. Under nutrient‐deprived condition, autophagy serves as an important energy production source, in which acidic hydrolases in autolysosomes degrade the cargoes sequestered by autophagosomes, releasing free amino acids.^4^ In this capacity and through its crosstalk with other signalling pathways, autophagy acts as a crucial pro‐survival mechanism, especially in face of cellular stress. Autophagy is also a potent suppressor of inflammation by repressing the p62/SQSTM1‐nuclear factor‐κB axis and inflammasome activation.^5^, ^6^ Many upstream signalling pathways or mediators, such as phosphoinositide‐3‐kinase (PI3K)‐Akt‐mammalian target of rapamycin (mTOR), AMP‐activated protein kinase (AMPK) and p53, have been shown to regulate autophagy 4. In this regard, the transcription factor EB (TFEB) was recently identified as a master coordinator of a transcriptional program that controls major autophagic steps by driving expression of autophagic and lysosomal genes.^7^ In the liver, autophagy not only regulates lipid metabolism and insulin resistance, but also protects hepatocytes from injury and cell death. Our research team and other investigators have reported that the lysosomal clearance of autophagosomes is defective in NAFLD, as a result of reduced lysosome acidification and lysosomal protease activity. Such autophagic flux impairment then exacerbates steatosis, insulin resistance and inflammation in NAFLD, through increasing endoplasmic reticulum stress.^8^, ^9^

Polydatin is a monocrystalline compound derived from the plant *Polygonum cuspidatum* Sieb. et Zucc., which is a traditional Chinese medicine commonly used for analgesic and diuretic purposes.^12^ Polydatin is also commonly detected in grapes, peanuts, hop cones and red wine, and is a natural precursor of resveratrol. The beneficial effects of polydatin have been widely reported, including neuroprotective activity in cerebral ischaemia,^13^ anti‐atherosclerotic effect in dyslipidemia^14^ and anti‐inflammatory effect in chronic lung diseases.^15^ Additionally, polydatin has been shown to protect against liver damage induced by alcohol,^16^ carbon tetrachloride^17^ and galactose/fructose overload.^18^, ^19^ A study also reported that polydatin can alleviate high‐fat diet‐induced NAFLD in rats.^20^ Nevertheless, the mechanism by which polydatin mediates its protective effects in NAFLD/NASH remains elusive. In this study, we examined if polydatin could exert its protective effect in a murine model of NASH and a cell line model of steatosis through rectifying the autophago‐lysosomal defect. Our results indicate that polydatin could up‐regulate the expression and activity of TFEB to restore autophagic flux in both models.

## 2 MATERIAL AND METHODS

### 2.1 Hepatocyte culture

The human hepatocyte cell line LO2 was obtained from the American Type Culture Collection (ATCC). LO2 cells were cultured in Dulbecco's modified Eagle's medium, supplemented with 10% foetal bovine serum and 1% penicillin‐streptomycin at 37°C in 5% CO_2_.

### 2.2 Western blots

Cells or tissues were harvested and washed with ice‐cold phosphate‐buffered saline (PBS), and lysed in immunoprecipitation assay buffer [150 mmol/L NaCl, 50 mmol/L Tris, 2 mmol/L ethyleneglycol‐bis(β‐aminoethylether), 2 mmol/L EDTA, 25 mmol/L NaF, 25 mmol/L β‐glycerophosphate, 0.2% Triton X‐100, 0.3% Nonidet P‐40, and 0.1 mmol/L phenylmethylsulfonyl fluoride]. Cellular debris was pelleted by centrifugation at 13 000 *g* for 30 minutes at 4°C. The concentrations of the total lysate protein were measured using a standard Bradford assay (Bio‐Rad, San Diego, CA). For Western blots, 10 μg of protein from the total cell lysate was electrophoresed by SDS–PAGE. The proteins were then transferred to nitrocellulose membrane (Pierce Chemical) and probed with primary antibodies followed by horseradish peroxidase‐labelled secondary antibodies. Proteins were visualized using enhanced chemiluminescence (Pierce Chemical).

### 2.3 Autophagic flux

LO2 cells were grown on glass chamber slides overnight and then transfected with mCherry‐GFP‐LC3 plasmid for 24 hours. After transfection, cells were treated with rapamycin (1.1 μmol/L), bafilomycin A1 (200 µmol/L), palmitic acid (60 µg/mL) or subject to serum starvation in the absence or presence of polydatin (24 μmol/L). Afterwards, cells were washed twice with PBS and fixed in 4% paraformaldehyde for 15 minutes at room temperature. After rinsing twice with PBS, the slides were mounted in ProLong Gold Anti‐fade reagent (Invitrogen, Carlsbad, CA, USA) and then examined under a confocal microscope (Leica).

### 2.4 Reverse transcription‐quantitative PCR

Total RNA was extracted by Trizol and then reverse‐transcribed into complementary DNA by a PrimeScript^TM^ RT reagent kit (TakaRa). mRNA expression of *TFEB* and its downstream genes was measured by quantitative PCR with SYBR Pre‐mix Ex Taq kit (TakaRa) using the following human primers: *TFEB*, forward: CAAGGCCAATGACCTGGAC, reverse: AGCTCCCTGGACTTTTGCAG; *PPP3CA*, forward: GCTGCCCTGATGAACCAAC, reverse: GCAGGTGGTTCTTTGAATCGG; *DPP7*, forward: GATTCGGAGGAACCTGAGTG, reverse: CGGAAGCAGGATCTTCTGG; *TPP1*, forward: GATCCCAGCTCTCCTCAATAC, reverse: GCCATTTTTGCACCGTGTG; *CTSF*, forward: ACAGAGGAGGAGTTCCGCACTA, reverse: GCTTGCTTCATCTTGTTGCCA; *CTSB*, forward: AGTGGAGAATGGCACACCCTA, reverse: AAGAGCCATTGTCACCC; *MITF*, forward: GGCTTGATGGATCCTGCTTTGC, reverse: GAAGGTTGGCTGGACAGGAGTT; *TFE3*, forward: GATCATCAGCCTGGAGTCCAGT, reverse: AGCAGATTCCCTGACACAGGCA. Mouse primers: *CTSB*, forward; TTAGCGCTCTCACTTCCACTACC, reverse: TGCTTGCTACCTTCCTCTGGTTA; *ATP6V0D1*, forward: GCATCTCAGAGCAGGACCTTGA, reverse: GGATAGGACACATGGCATCAGC; *TcFEB*, forward: GCGAGAGCTAACAGATGCTGA, reverse: CCGGTCATTGATGTTGAACC; *PPP3CA*, forward: ATCCCAAGTTGTCGACGACC, reverse: ACACTTTCTTCCAGCCTGCC; *MCOLN1*, forward: GCGCCTATGACACCATCAA, reverse: TATCCTGGACTGCTCGAT; *HPRT*, forward: CAAGCTTGCTGGTGAAAAGG, reverse: GTCAAGGGCATATCCAACAAC; *MITF*, forward: GATCGACCTCTACAGCAACCAG, reverse: GCTCTTGCTTCAGACTCTGTGG; *TFE3*, forward: CTATCTTCCAGGAGGCACTGCA, reverse: CTCGCGTTTGATGTTAGGCAGC. Endogenous control 28S was used. The relative mRNA expression levels were calculated by the double delta C_T_ method.

### 2.5 Biochemical analysis

Serum alanine transaminase (ALT) levels, serum cholesterol levels and serum triglyceride levels were determined using Vet Test Chemistry Analyzer (IDEXX) according to the manufacturer's instructions. Hepatic total triglyceride levels were detected using the Triglyceride assay kit (ab65336). Hepatic total cholesterol levels were detected using the Cholesterol/Cholesteryl Ester Assay Kit (ab65369).

### 2.6 Animal experiment

Age‐matched C57Bl/KsJ‐db‐/db (*db/db*) mice were fed either methionine‐choline deficient (MCD) or control diet for 4 weeks. Polydatin (100 mg/kg) was administered by oral gavage every other day. All procedures were approved by the Animal Experimentation Ethics Committee of the Chinese University of Hong Kong.

### 2.7 Oil red O staining

Cells were exposed to palmitic acid in the absence or presence of polydatin (24 μmol/L) for 24 hours. Afterwards, cells were rinsed three times with PBS and fixed in 10% formalin for 1 hour. The fixed cells were washed by PBS three times and stained with Oil Red O solution (stock solution, 0.5 g/mL in isopropanol; working solution, 30 mL of Oil Red O stock solution and 20 mL of distilled water) for 15 minutes. After staining, the unbound dye was washed by 60% isopropanol. Nuclei were slightly stained by haematoxylin for 5 seconds. Oil Red O content levels in tissues were calculated by dissolving the Oil red O dye in isopropanol and measured the absorbance at 510 nm. 100% isopropanol was used as blank. For in vivo study, 10 μm‐frozen sections of livers were cut and fixed in 10% formalin and followed by Oil Red O staining as described for in vitro staining.

### 2.8 Nuclear/cytosolic fractionation

Cells at 70% of confluence in six well dishes were exposed to palmitic acid in the absence or presence of polydatin (24 μmol/L) or treated with polydatin (24 μmol/L) alone for 24 hours. Afterwards, cells were rinsed three times with PBS and were subject to subcellular fractionation. In brief, cells were lysed in hypotonic lysis buffer (100 mmol/L HEPES, pH 7.9 with 15 mmol/L MgCl_2_, 100 mmol/L KCl and 10% NP‐40 supplemented with 0.1 mol/L dithiothreitol solution as well as protease inhibitor cocktail) on ice for 15 minutes. After 30 seconds, the lysate was centrifugated. The supernatant and pellet represented cytosolic fraction and nuclear fraction, respectively. Afterwards, nuclear faction was washed twice and lysed in extraction buffer (20 mmol/L HEPES, pH 7.9 with 1.5 mmol/L MgCl_2_, 0.42M NaCl, 0.2 mmol/L EDTA and 25% (v/v) glycerol) containing dithiothreitol solution and protease inhibitor cocktail and subsequently was centrifuged at 21 000 *g* for 5 minutes.

### 2.9 Lysosome enzyme activities

Tissues were harvested and lysed in immunoprecipitation assay buffer. Cellular debris was pelleted by centrifugation at 13 000 *g* for 30 minutes at 4°C. The total lysate protein were used to detect lysosomal enzyme activities using the Acid Phosphatase Assay Kit (Catalog Number CS0740; Sigma‐Aldrich), the β‐N‐Acetylglucosaminidase Assay Kit (Catalog Number CS0780; Sigma‐Aldrich) and Cathepsin D Activity Assay Kit (ab65302; Abcam).

### 2.10 Histological analyses

The 5‐μm thick paraffin sections were stained with haematoxylin and eosin and then were rated by NAFLD scores as previously described.^21^ In general, the NAFLD scores consist of three features, namely steatosis, lobular inflammation and hepatocellular ballooning. The scores were rated by two pathologists.

### 2.11 Histology and immunohistochemical staining

Immunohistochemistry of p62 and LC3 was performed on 5‐μm thick paraffin sections. The paraffin sections were preheated in a microwave oven for 10 minutes and were blocked with bovine serum albumin (1:200). Afterwards, the sections were incubated with anti‐p62 antibody (1:1000; BD Biosciences) and anti‐LC3 (1:1000; Novus) for the mouse liver sections. After primary antibody staining, peroxidase‐labelled polymer were used for signal detection and the sections were subsequently counterstained with haematoxylin for the nuclei. For semi‐quantitative analysis of p62 and LC3 accumulation, the scores were rated as grades 0 (none), 1 (minor), 2 (moderate) and 3 (severe). More than 10 sections in each mouse were evaluated. The scores were rated by two pathologists.

### 2.12 Statistical analysis

Statistical analysis was performed with one‐way analysis of variance (ANOVA) followed by the Tukey's post hoc test. *P* < 0.05 were considered statistically significant.

## 3 RESULTS

### 3.1 Polydatin ameliorates murine steatohepatitis in vivo and hepatic steatosis in vitro

**Figure 1:**
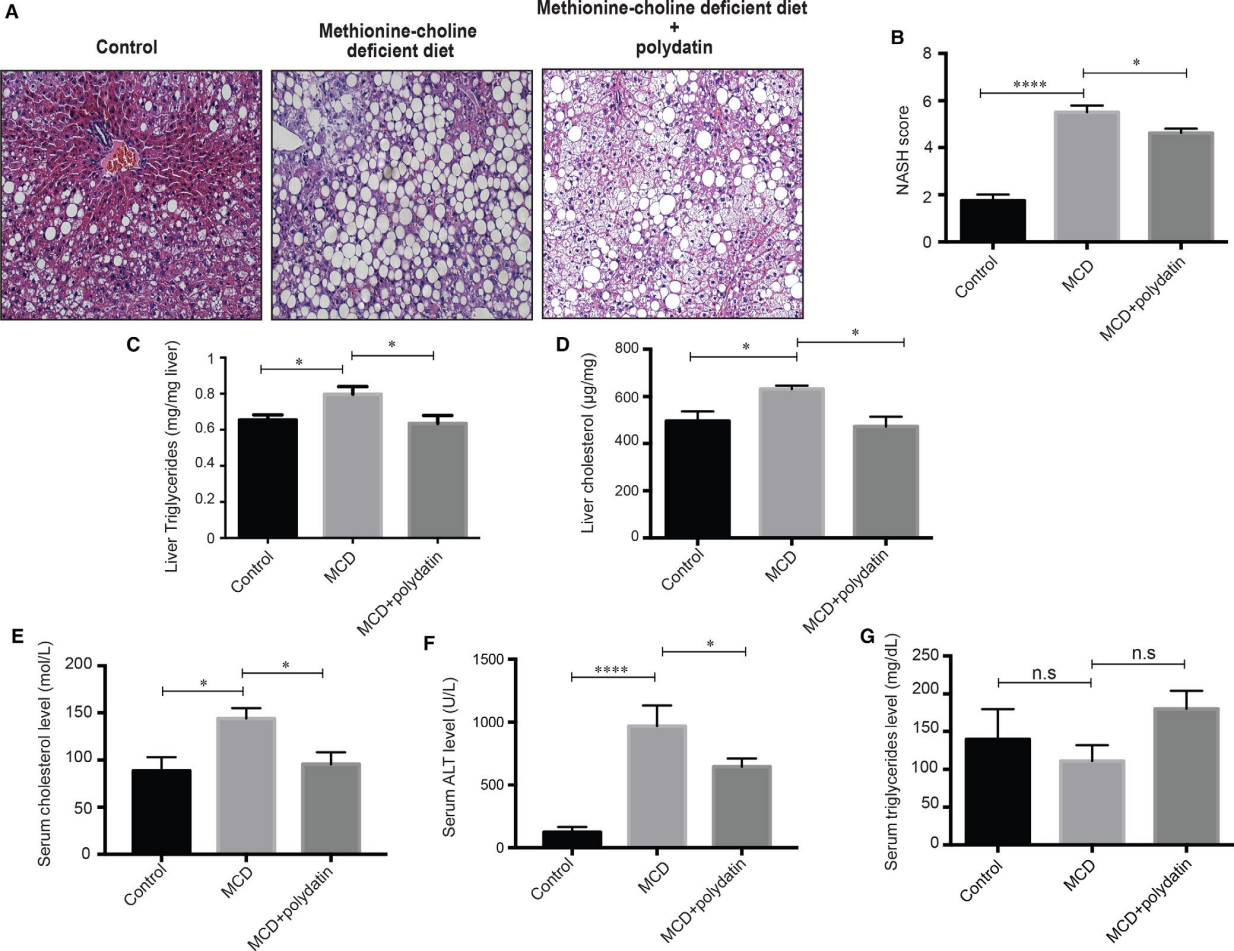
Effects of polydatin on liver of methionine‐choline deficient (MCD) diet‐fed *db/db* mice. Polydatin ameliorated MCD diet‐induced hepatic damages. (A) Representative H&E staining from *db/db* mice fed control diet, MCD diet, or MCD diet with polydatin. Polydatin was administered by oral gavage at the dosage of 100 mg/kg every other day for 4 wk. Bars = 50 µm; (B) Non‐alcoholic steatohepatitis (NASH) activity score calculated for steatosis, lobular inflammation and ballooning; (C‐D) The content of liver triglyceride and hepatic total cholesterol from liver tissues were measured; (E‐G) Serum total cholesterol, aminotransferases (ALT) levels and triglycerides levels of mice. Mean ± SEM (n = 8 per group). *P < 0.05; ****P < 0.0001

**Figure 2:**
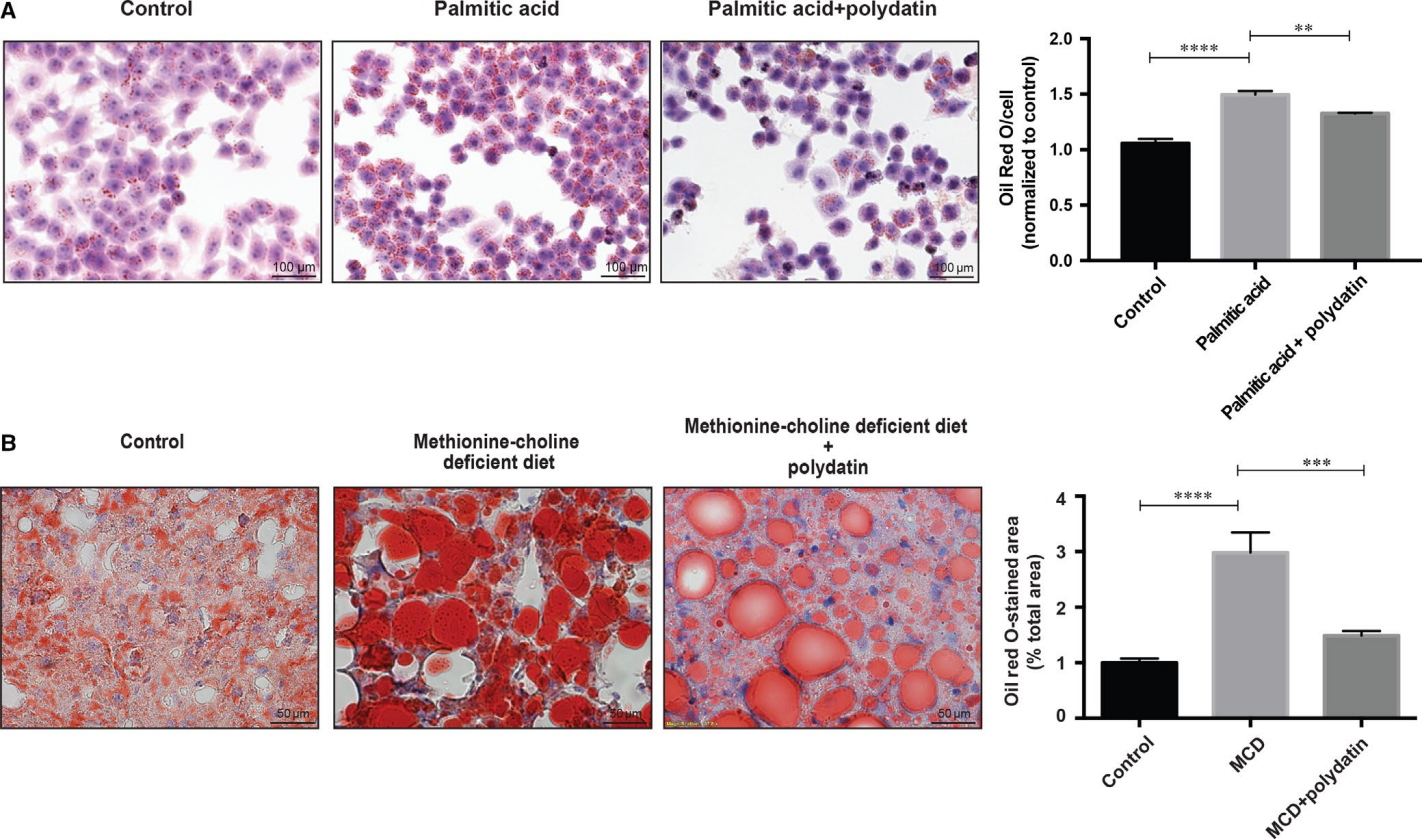
Effects of polydatin on lipid accumulation in LO2 hepatocytes and methionine‐choline deficient (MCD) diet‐fed *db/db* mice. (A) Treatment with polydatin at the concentration of 24 μmol/L for 24 h alleviated palmitic acid (PA; 60 µg/mL)‐induced lipid accumulation. Representative Oil‐Red‐O staining of hepatocytes from different groups were shown. Oil Red O contents from different groups were determined by colorimetry. Mean ± SEM (n = 3). **P < 0.01; ****P < 0.0001. (B) Representative Oil red O staining from *db/db* mice fed control diet, MCD diet, or MCD diet with polydatin. Bars = 50 µm. Mean ± SEM (n = 8 per group). ***P < 0.001; ****P < 0.0001

To elucidate the action of polydatin in the development of steatohepatitis, we first used a genetic mutation model (*db/db* mice), which has a point mutation in leptin receptor, and fed MCD diet to mimic the pathogenesis and aetiology of human NASH.^22^, ^23^ We demonstrated that oral gavage of polydatin (100 mg/kg) every other day for 4 weeks reduced NAFLD activity score 21, 24 (Figure 1A,B) as well as liver levels of triglyceride (Figure 1C) accompanied by reduced liver levels of cholesterol (Figure 1D). Besides, polydatin attenuated serum levels of cholesterol (Figure 1E) and alanine aminotransferase (ALT) (Figure 1F) without significant effect on serum levels of triglycerides (Figure 1G). Apart from NASH animal model, we cultured human hepatocyte LO2 cells with palmitic acid to trigger lipotoxic condition in vitro. Plasma levels of palmitic acid, which is a saturated fatty acid, has been reported to be elevated in patients with NASH and induce lipo‐toxicity in hepatocytes.^25^, ^26^ Our results demonstrated that polydatin reduced lipid accumulation in palmitic acid‐exposed human hepatic LO2 cells and a murine NASH model as shown by Oil Red O staining (Figure 2A,B).

### 3.2 Polydatin restores impaired autophagic flux in vivo and in vitro

**Figure 3:**
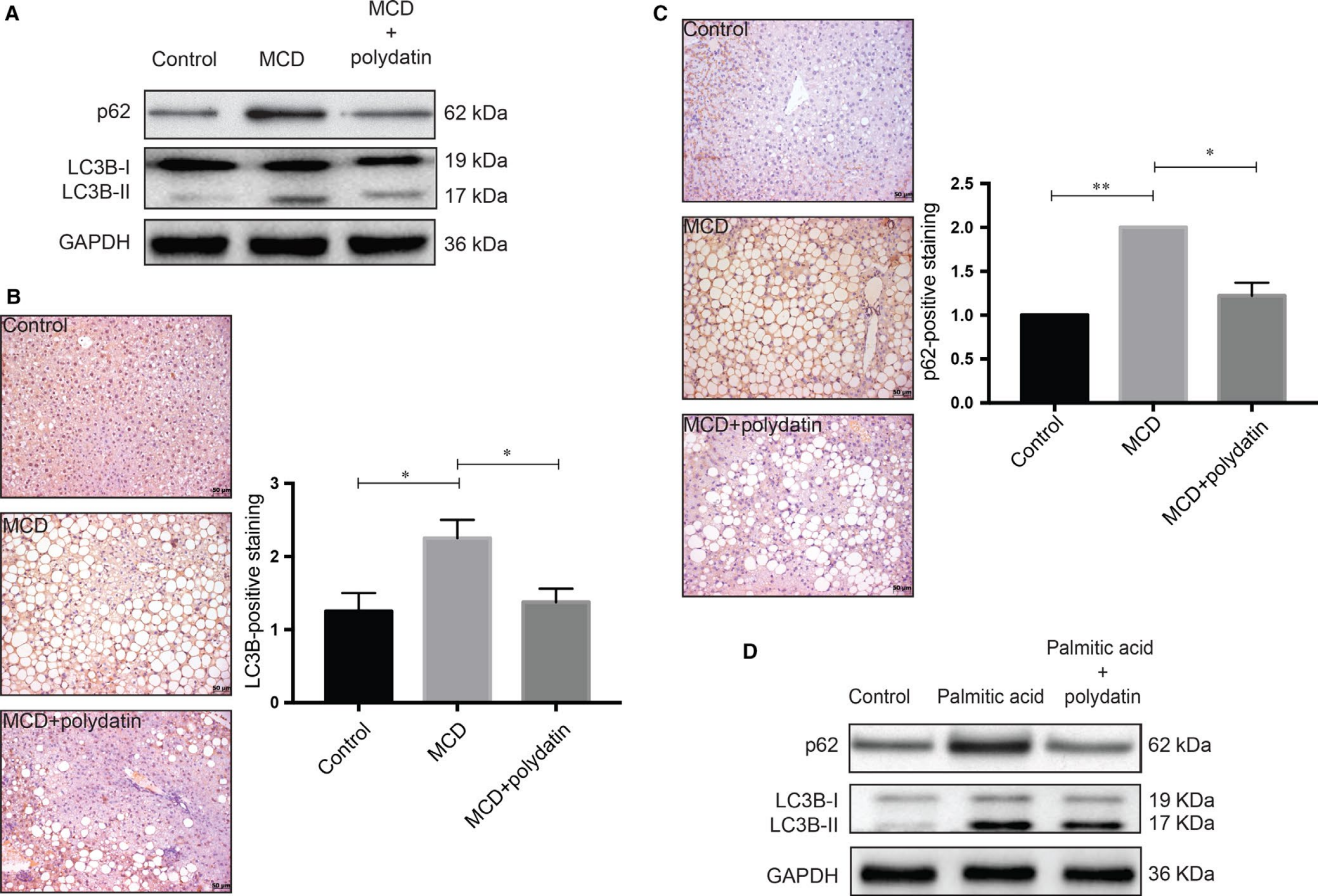
Alleviation of steatosis/steatohepatitis‐induced impairment of autophagic flux by polydatin. Polydatin rectified the autophagic impairment as shown by reduced accumulation of LC3B‐II and p62/SQSTM1. (A) Protein levels of autophagy markers (LC3B and p62) in non‐alcoholic steatohepatitis (NASH) mouse liver samples were determined. Polydatin administered at the dosage of 100 mg/kg every other day for 4 weeks reduced the hepatic accumulation of LC3B‐II and p62 in NASH mice. Representative blots from three independent experiments were shown. (B and C) Immunohistochemical assessment of autophagy markers p62 and LC3B in liver tissues was performed. Liver tissue sections were stained using LC3B or p62 antibodies. For semi‐quantitative analysis of p62 and LC3 accumulation, the scores were rated as grades 0 (none), 1 (minor), 2 (moderate) and 3 (severe). More than 10 sections in each mouse were evaluated. (D) Protein levels of autophagy markers in palmitic acid (PA; 60 µg/mL)‐exposed human LO2 cells were measured. Polydatin (24 μmol/L) diminished the concomitant accumulation of LC3B‐II and p62 caused by PA exposure (24 h). Representative blots were selected from three independent experiments. Bars = 50 µm. Mean ± SEM. *P < 0.05; **P < 0.01

To further explore the effect of polydatin on autophagy, we first determined the protein levels of autophagosome marker LC3B‐II and the autophagic substrate SQSTM1/p62 in our murine NASH model. MCD diet resulted in increases in both LC3B‐II and SQSTM1/p62 protein levels in *db/db* mice, suggesting a blockade of autophago‐lysosomal degradation function. Notably, we observed that both LC3B‐II and SQSTM1/p62 protein levels were lowered by polydatin treatment in our mouse NASH model, suggesting polydatin could rectify the impaired lysosomal degradation function (Figure 3A).

As accumulation of LC3B and p62 is a landmark characteristics of autophagic impairment in NASH, we performed immunohistochemistry staining of these two autophagy markers in liver tissues. Liver sections from animals fed MCD diet demonstrated concomitant accumulation of LC3B and p62. Strikingly, both immunoreactivities were attenuated by the administration of polydatin as shown in Figure 3B,C and Figure S1B. Consistent with the in vivo model, we observed concomitant increases in LC3B‐II and SQSTM1/p62 protein levels in palmitic acid‐exposed cells, whereas these increases were reversed by polydatin treatment (Figure 3D), indicating that polydatin could alleviate palmitic acid‐triggered impaired autophagic flux. The effects of polydatin alone on LC3B‐II and SQSTM1/p62 protein levels in LO2 cells were shown in the left panel of Figure S1A. These findings suggested the mechanistic action of polydatin in restoring autophagic flux in NASH, presumably by promoting autophagosome–lysosome fusion or increasing the lysosome‐dependent degradation.

### 3.3 Polydatin reactivates lysosomal activity in vivo and in vitro

**Figure 4:**
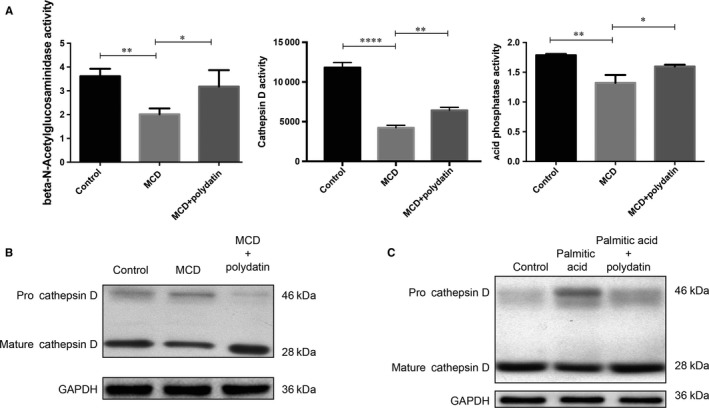
Reactivation of lysosomal enzyme activities by polydatin. Polydatin alleviated methionine‐choline deficient diet‐triggered and PA‐induced pro‐cathepsin D accumulation and reduction of lysosomal enzyme activities. (A) Hepatic lysosomal enzyme activities (β‐N‐acetylglucoseaminidase, acid phosphatase, and cathepsin (D) were measured. (B) Hepatic levels of pro‐cathepsin D and mature cathepsin D from non‐alcoholic steatohepatitis mouse model were determined by Western blots. (C) LO2 cells were exposed to palmitic acid (60 µg/mL) for 24 h in the absence or presence of polydatin (24 µmol/L). Pro‐cathepsin D and mature cathepsin levels were determined by Western blots. Representative blots were selected from three independent experiments. Mean ± SEM. *P < 0.05; **P < 0.01; ****P < 0.0001

To evaluate the impact of polydatin on lysosomal function, we measured three lysosomal enzyme activities, namely acid phosphatase,^27^ N‐acetyl‐β‐D‐glucosaminidase (β‐NAG)^28^ and cathepsin D 29 in the liver of mouse NASH model. MCD diet markedly reduced the activities of all three enzymes compared with the control diet group, whereas normalized activities were observed after polydatin treatment (Figure 4A). Conversion of cathepsin D protein from its inactive precursor to the mature form, which depends on intact lysosome acidification, is considered one of the hallmarks of lysosomal maturation and degradative function.^30^ As shown in Figure 4B,C, both MCD diet in vivo and palmitic acid in vitro caused a stagnant conversion of cathepsin D protein from its precursor into the mature form, which was also reversed by polydatin. Polydatin per se in the absence of palmitic acid did not significantly affect pro‐ and mature cathepsin D levels in LO2 cells (right panel, Figure S1A).

### 3.4 Polydatin restores autophagosome‐lysosome acidification

**Figure 5:**
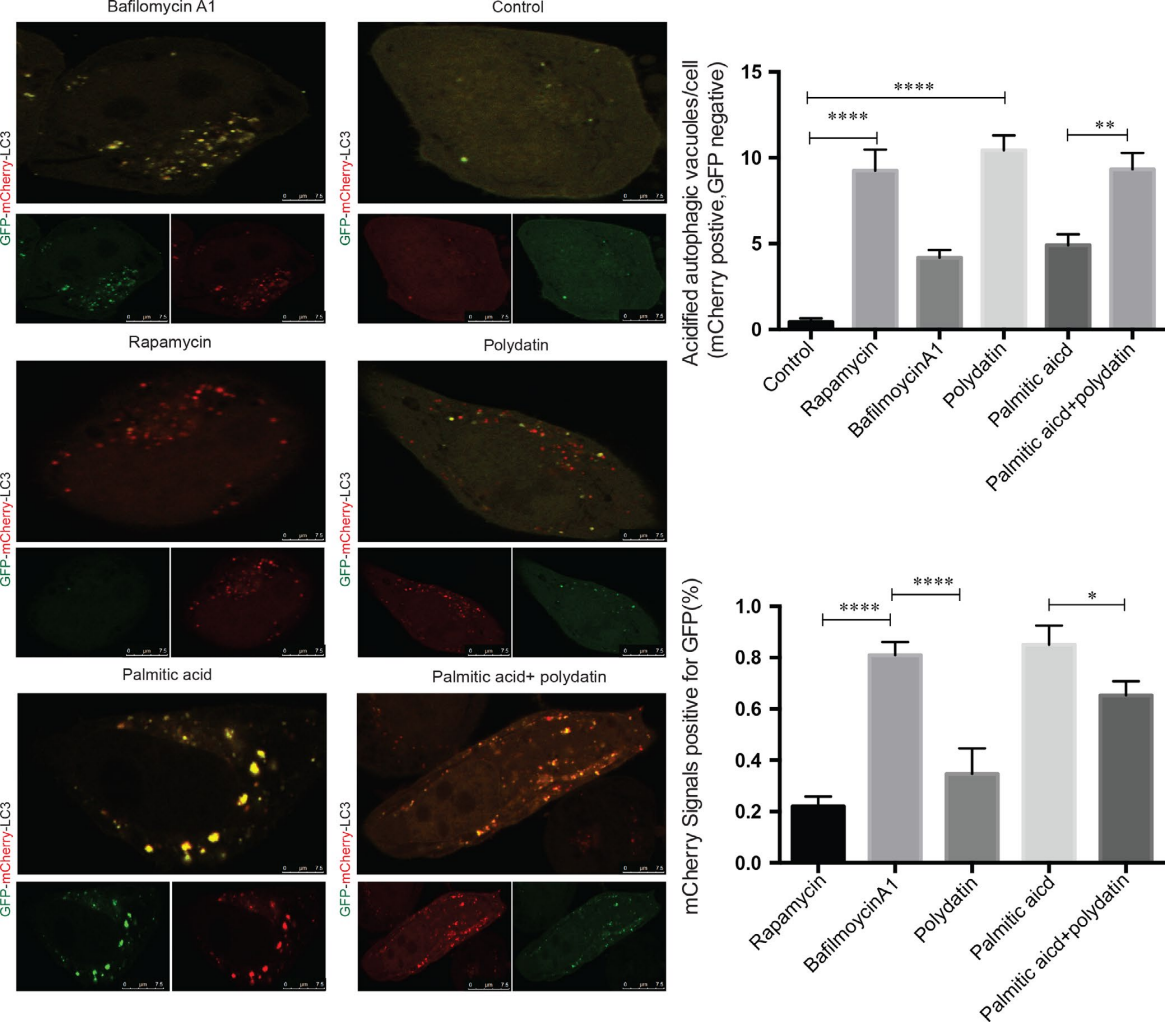
Rectification of palmitic acid‐induced impairment of autolysosomal acidification by polydatin in LO2 hepatocytes. LO2 cells were transfected with mCherry‐GFP‐LC3 plasmid for 24 h followed by treatment with rapamycin (1.1 µmol/L), polydatin (24 µmol/L), bafilomycin A1 (200 µmol/L) or palmitic acid (60 µg/mL) in the absence or presence of polydatin (24 µmol/L) for additional 24 h. Acidified and non‐acidified LC3‐positive autophagosomes were visualized and counted under a confocal microscope. Mean ± SEM in three independent experiments.*P < 0.05; **P < 0.01; ****P < 0.0001

To further understand the mechanism by which polydatin restored autophagic flux, LO2 hepatocytes were transfected with the plasmid GFP‐mCherry‐LC3B to assay the acidification of autophagosomes. The GFP‐mCherry‐LC3B comprises of an acid‐sensitive GFP mutant and an acid‐insensitive mCherry mutant, which fluoresces red dots (mCherry positive and GFP negative) at acidic pH to represent acidic LC3B‐positive autolysosomes and fluoresces yellow dots in neutral pH to represent non‐acidic autophagosomes.^30^, ^31^ Using this approach, we observed a retained GFP fluorescence co‐localized with mCherry signals in palmitic acid‐incubated LO2 hepatocytes, suggesting the accumulation of non‐acidic autophagosomes. On the contrary, in polydatin‐treated LO2 hepatocytes, most of the LC3B‐positive autophagic puncta lost GFP signal but retained mCherry signal, indicating unimpeded autophagosomal degradation (Figure 5). Bafilomycin A1 and rapamycin, which are the classical autophagy inhibitor and inducer respectively, were also included as control in this set of experiments. In this regard, bafilomycin A1 mimicked the effect of palmitic acid by causing accumulation of non‐acidic autophagosomes (Figure 5). These findings indicated that polydatin rectified the palmitic acid‐induced autophagic flux impairment by enhancing the formation of acidic autolysosomes.

### 3.5 Polydatin activates tfeb for lysosome biogenesis

**Figure 6:**
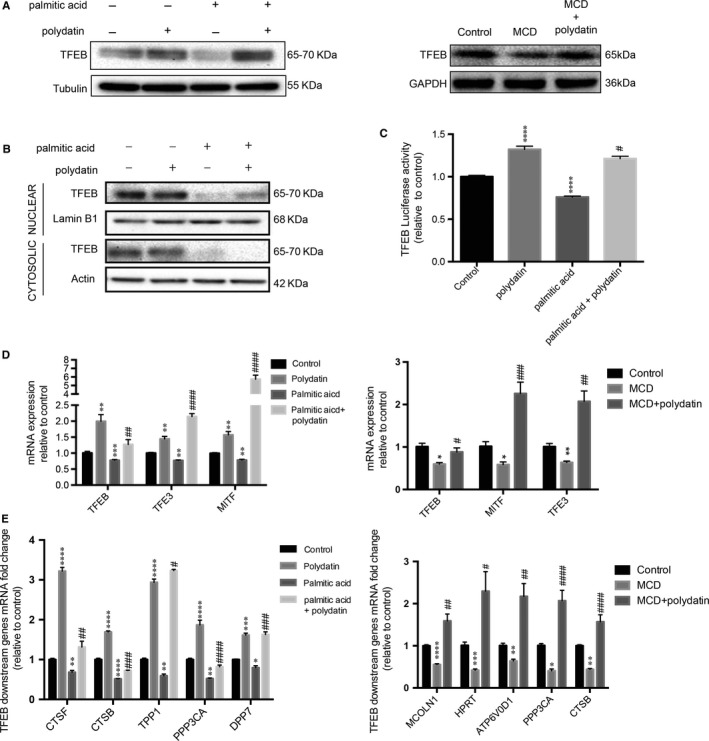
Restoration of transcription factor EB (TFEB) transcription activity, mRNA and protein levels by polydatin. (A) LO2 cells were treated with polydatin (24 µmol/L) alone or exposed to palmitic acid (PA; 60 µg/mL) for 24 h in the absence or presence of polydatin (24 µmol/L). Hepatic tissues were extracted from non‐alcoholic steatohepatitis (NASH) mouse model. TFEB levels were determined by Western blots. Representative blots were selected from three independent experiments. (B) The cytosolic and nuclear fractions were isolated and the expression levels of TFEB were evaluated by Western blots. Representative blots were selected from three independent experiments. (C) LO2 cells were transfected with vector only or 4X CLEAR (Coordinated Lysosomal Expression and Regulation) promoter–luciferase vector and exposed to polydatin (24 µmol/L) alone or PA (60 µg/mL) in the absence or presence of polydatin (24 µmol/L) for 24 h. Afterwards, luciferase activity of TFEB was assayed. (D) Relative quantitative PCR analysis of mRNA levels of MiTF/TFE family members in LO2 cells treated with polydatin (24 µmol/L) alone or exposed to palmitic acid (60 µg/mL) in the absence or presence of polydatin (24 µmol/L) for 24 h or in hepatic tissues of NASH mouse model. (E) Relative quantitative PCR analysis of mRNA levels of TFEB target genes in LO2 cells treated with polydatin (24 µmol/L) alone or exposed to PA (60 µg/mL) in the absence or presence of polydatin (24 µmol/L) for 24 h and in hepatic tissues of NASH mouse model. Mean ± SEM in three independent experiments. *P < 0.05; **P < 0.01; ***P < 0.001;****P < 0.0001 when comparing control and polydatin or PA; comparing control diet and methionine‐choline deficient (MCD) diet. ^#^ *P* < 0.05; ^##^ *P* < 0.01; ^###^ *P* < 0.001; ^####^ *P* < 0.0001 when comparing PA and the combination of PA and polydatin; comparing MCD diet and MCD diet with the administration of polydatin

Given the central role of TFEB in the regulation of lysosome biogenesis,^7^, ^32^ we next determined whether this transcription factor was involved in polydatin‐induced restoration of lysosome function in NASH. Herein, we demonstrated that LO2 challenged with palmitic acid showed a significant decrease in TFEB protein levels and this reduction could be alleviated upon treatment with polydatin (left panel, Figure 6A). Consistent with the in vitro data, we observed that polydatin restored TFEB protein levels in MCD diet‐induced NASH in mice (right panel, Figure 6A). Besides, we demonstrated that polydatin enhanced TFEB nuclear translocation in LO2 cells challenged with palmitic acid as shown in Figure 6B. To further confirm the changes of transcriptional activity of TFEB, we transfected 4× CLEAR (Coordinated Lysosomal Expression and Regulation) promoter luciferase reporter into LO2 cells, followed by the exposure to palmitic acid alone or in combination with polydatin. We observed remarkable decreases in mRNA levels of MiTF/TFE family of basic helix‐loop‐helix leucine zipper transcription factors, such as TFEB, TFE3, MITF and transcriptional activity of TFEB by palmitic acid, both of which were restored by polydatin (Figure 6C and left panel, Figure 6D). Consistently, we demonstrated that polydatin restored MiTF/TFE family (TFEB, TFE3, MITF) mRNA levels in MCD diet‐induced NASH in mice (right panel, Figure 6D). Moreover, the mRNA levels of multiple known TFEB target genes, namely dipeptidyl peptidase 7 (*DDP7*), tripeptidyl‐peptidase I (*TPP1*), protein phosphatase 3 catalytic subunit alpha (*PPP3CA*), cathepsin B (*CTSB*) and cathepsin F (*CTSF*) were restored by polydatin in palmitic acid‐exposed LO2 cells (left panel, Figure 6E). In line with the in vitro data, the mRNA levels of multiple known TFEB target gens, namely mucolipin‐1 (MCOLN1), hypoxanthine‐guanine phosphoribosyl‐transferase (HPRT), v‐type proton ATPase subunit d1 (ATP6V0D1), PPP3CA and CTSB were rectified by the administration of polydatin in MCD‐diet‐induced NASH in mice. (Figure 6F right panel).

### 3.6 Polydatin inhibits mTOR signalling

**Figure 7:**
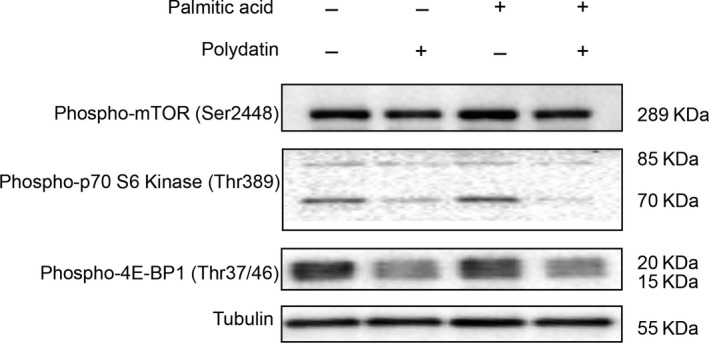
Inhibition of mTOR pathway by polydatin in LO2 hepatocytes. Polydatin‐enhanced autophagic flux was accompanied by inhibition of mTOR. LO2 cells were treated with polydatin (24 µmol/L) alone or exposed to palmitic acid (PA; 60 µg/mL) for 24 h in the absence or presence of polydatin (24 µmol/L). Phosphorylation levels of mTOR (Ser2448) or its related proteins p70‐S6K (Thr389) and 4E‐BP1 (Thr37/46) were determined by Western blots. Representative blots of three independent experiments are shown

mTOR signalling is known to negatively regulate TFEB and autophagy. To explore if polydatin‐enhanced autophagic flux was associated with the inhibition of mTOR signalling, we determined the phosphorylation levels of mTOR (Ser2448) and its downstream substrates p70S6K (Thr389) and 4E‐BP1 (Thr37/46). Results showed that polydatin reduced phosphorylation levels of all the three proteins (Figure 7), suggesting that polydatin‐regulated autophagy was associated with inhibition of the mTOR pathway.

## 4 DISCUSSION

Polydatin is a natural precursor of resveratrol with antioxidant and anti‐inflammatory activities. A recent study reported that polydatin could alleviate insulin resistance and hepatic steatosis in rats.^20^ Besides, evidence showed that polydatin could improve glucose and lipid metabolisms in hepatocytes cells through the AMPK pathway.^33^ However, the cellular and molecular mechanism by which polydatin protects against NASH is still ambiguous. In this study, for the first time, we found that polydatin‐ameliorated NASH was associated with activation of TFEB‐mediated lysosomal clearance of autophagosomes.

Our recent study suggests that autophagy is impaired in NAFLD/NASH as a result of defective lysosome acidification.^9^ MiT‐TFE family transcription factors, including TFEB, TFE3 and MITF, contribute to modulation of lysosome biogenesis, cellular energy homeostasis, and regulation of autophagy.^34^ Among these transcriptional factors, TFEB is a master regulator of autophagy by promoting lysosomal biogenesis and functions 35 and therefore could be a promising therapeutic target. Under the condition of starvation, TFEB is phosphorylated by ERK and translocated into the nucleus, and eventually activates autophagy.^7^ TFEB has also been linked to lipid metabolism and oxidative stress in hepatocytes,^32^, ^36^ both of which are crucial factors in the development of liver diseases, including NASH.^37^ Therefore, pharmacological activation of TFEB represents a rational approach. In this study, we found that palmitic acid‐induced and MCD diet‐triggered suppression of MiT‐TFE transcriptional factors was antagonized by polydatin.

Impaired autophagic flux may indicate defective lysosomal clearance of autophagosomes in the late phase of autophagy.^38^ In palmitic acid‐incubated hepatocytes, we observed that the impairment of autophagic flux was correlated with an dysfunctional lysosomal acidification and reduced activity of lysosomal enzymes, such as cathepsin D, presumably due to the reduction in TFEB expression and activity. Our data are concordant with two previous studies suggesting that palmitic acid could impair autophagic flux by enhancing ER stress 11 and deregulate lysosomal acidification and thereby blocking autophagy in hepatocytes.^9^ In this connection, polydatin restored autophagic flux by increasing TFEB transcription and activity and subsequently promoting cathepsin D activity. In the future, it is worthwhile to examine the potential connection between ER stress and TFEB function.

In this study, we examined the therapeutic effect of polydatin in the experimental steatohepatitis model using a nutritional model of NASH (ie *db/db* mice fed MCD diet), demonstrating that this phytochemical could reduce liver damage and lipid accumulation as evidenced by diminished NAFLD activity score, along with reduced serum cholesterol and ALT levels. Consistent with our study, another team showed that polydatin could ameliorate high‐fat diet‐induced NASH in terms of lipid peroxidation and inflammation.^39^ Our data further suggested that polydatin could exert its therapeutic effect in NASH by up‐regulating the transcription factor TFEB and subsequently restoring the lysosomal clearance of autophagosomes. Our study along with others collectively provided useful animal efficacy data and mechanistic insights to inform future clinical trials for examining the potential anti‐NASH effect of polydatin in humans.

## CONFLICT OF INTEREST

The authors confirm that there is no conflict of interest.
