# Supplementary figures and images for: The phytochemical polydatin ameliorates non‐alcoholic steatohepatitis by restoring lysosomal function and autophagic flux
Source: J Cell Mol Med. 2019 Apr 11;23(6):4290–300. doi: 10.1111/jcmm.14320 (PMC6533566; doi:10.1111/jcmm.14320)

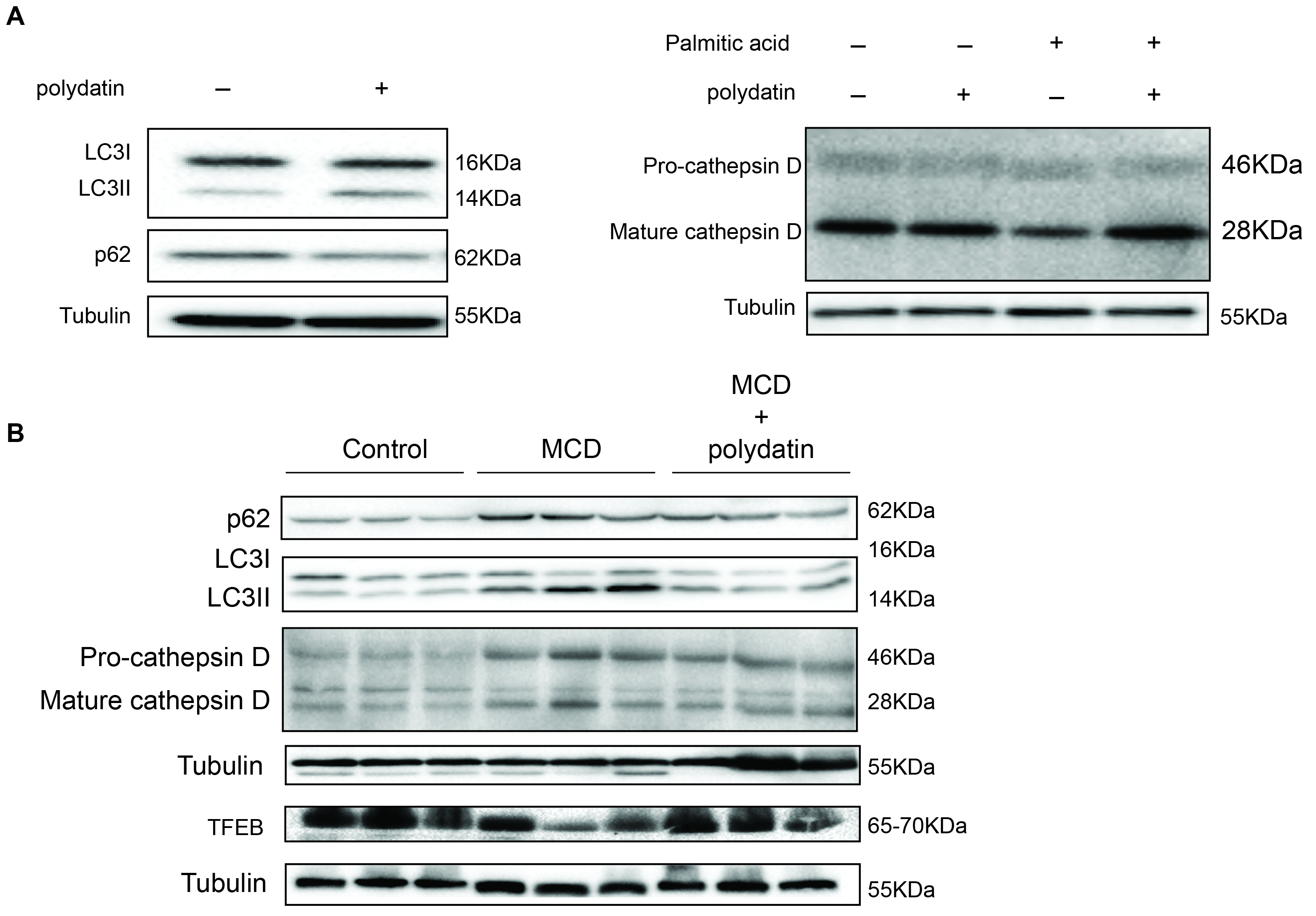

Supplement: Supplementary file 1 [file JCMM-23-4290-s001.tif]
